# Supplementary material for: The causal relationship between the human gut microbiota and pyogenic arthritis: a Mendelian randomization study
Source: Front Cell Infect Microbiol. 2024 Nov 26;14:1452480. doi: 10.3389/fcimb.2024.1452480 (PMC11629706; doi:10.3389/fcimb.2024.1452480)
Supplement: Supplementary file 1 [file DataSheet1.zip › mendelian test/ebi-a-GCST90027499.csv_forest.pdf]

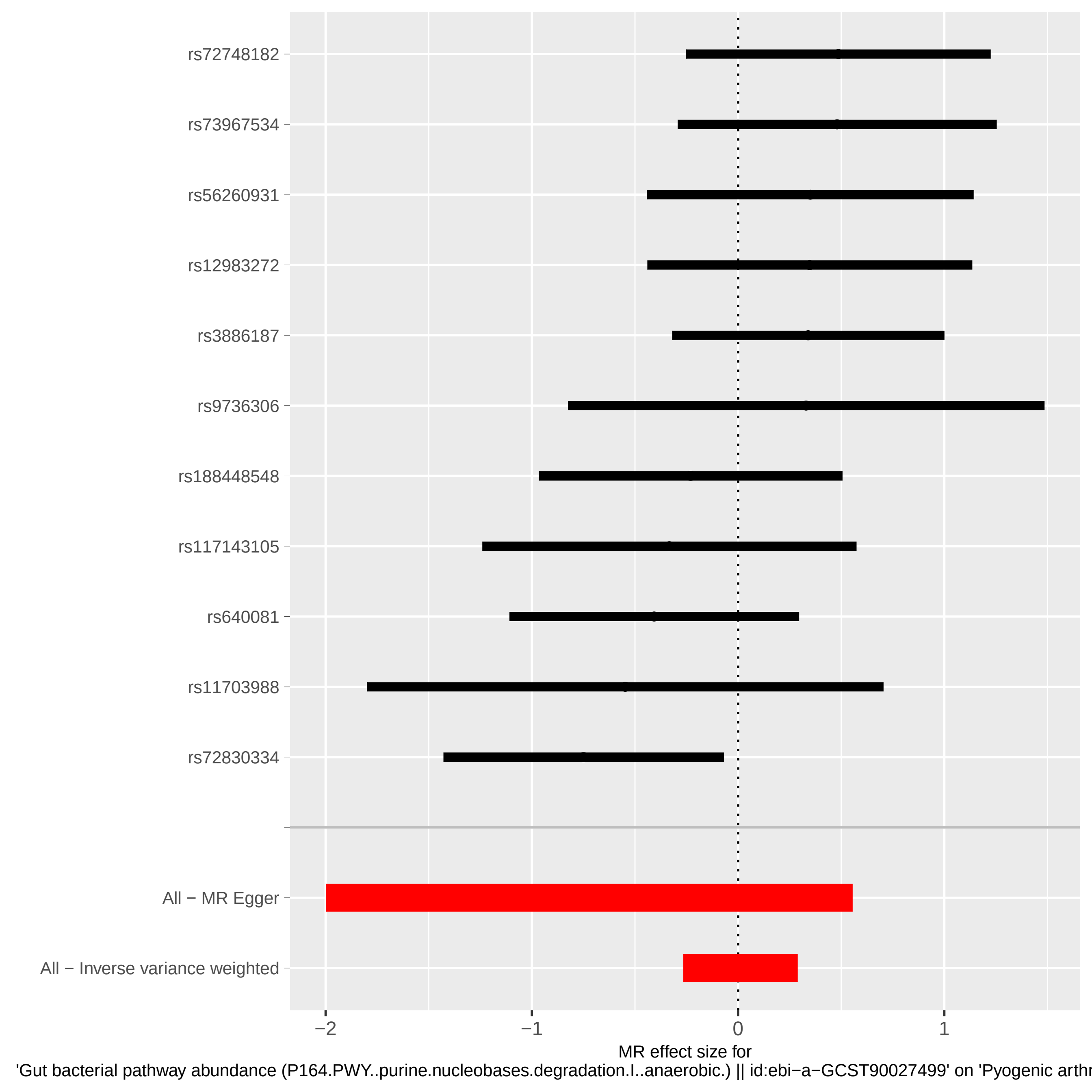
rs72748182
rs73967534
rs56260931
rs12983272
rs3886187
rs9736306
rs188448548
rs117143105
rs640081
rs11703988
rs72830334
All − MR Egger
All − Inverse variance weighted
−2
−1
0
1
MR effect size for
'Gut bacterial pathway abundance (P164.PWY..purine.nucleobases.degradation.I..anaerobic.) || id:ebi−a−GCST90027499' on 'Pyogenic arthr
